# Comprehensive Analysis of the Role of Gene Variants in Matrix Metalloproteinases and Their Tissue Inhibitors in Retinopathy of Prematurity: A Study in the Polish Population

**DOI:** 10.3390/ijms242015309

**Published:** 2023-10-18

**Authors:** Aneta Choręziak-Michalak, Dawid Szpecht, Anna Chmielarz-Czarnocińska, Agnieszka Seremak-Mrozikiewicz, Krzysztof Drews, Grażyna Kurzawińska, Ewa Strauss, Anna Gotz-Więckowska

**Affiliations:** 1 Department of Ophthalmology, Poznan University of Medical Sciences, ul. Augustyna Szamarzewskiego 84, 61-848 Poznan, Poland; anetachoreziak@gmail.com (A.C.-M.); anna.czarnocinska@ump.edu.pl (A.C.-C.); agotzwieckowska@ump.edu.pl (A.G.-W.); 2 Department of Neonatology, Poznan University of Medical Sciences, ul. Polna 33, 60-535 Poznan, Poland; dawidszpecht@ump.edu.pl; 3 Department of Perinatology and Women’s Diseases, Poznan University of Medical Sciences, ul. Polna 33, 60-535 Poznan, Poland; a.mrozikiewicz@ump.edu.pl (A.S.-M.); kdrews@ump.edu.pl (K.D.); gkurzawinska@ump.edu.pl (G.K.); 4 Institute of Human Genetics, Polish Academy of Sciences, ul. Strzeszynska 32, 60-479 Poznan, Poland

**Keywords:** retinopathy of prematurity, matrix metalloproteinases, MMP-1, MMP-9, tissue inhibitors of matrix metalloproteinases, prematurity

## Abstract

This study was designed to investigate the relationship between variants of matrix metalloproteinases (*MMP*-1 rs179975, *MMP*-9 rs17576 and rs17577), their tissue inhibitors (*TIMP*-1 rs4898, *TIMP*-2 rs2277698 and rs55743137) and the development of retinopathy of prematurity (ROP) in infants from the Polish population. A cohort of 100 premature infants (47% female) was enrolled, including 50 ROP cases and 50 no-ROP controls. Patients with ROP were divided into those with spontaneous remission and those requiring treatment. A positive association between *MMP*-1 rs179975 1G deletion allele and ROP was observed in the log-additive model (OR = 5.01; *p* = 0.048). Furthermore, female neonates were observed to have a negative association between the *TIMP-1* rs4898C allele and the occurrence of ROP and ROP requiring treatment (codominant models with respective *p*-values < 0.05 and 0.043). Two and three loci interactions between *MMP*-1 rs1799750 and *TIMP1rs4989* (*p* = 0.015), as well as *MMP*-1 rs1799750, *MMP*-9 rs17576 and *TIMP*-*1* rs4989 (*p* = 0.0003) variants influencing the ROP risk were also observed. In conclusion, these findings suggest a potential role of *MMPs* and *TIMPs* genetic variations in the development of ROP in the Polish population. Further studies using a larger group of premature infants will be required for validation.

## 1. Introduction

On a global scale, the prevalence of retinopathy of prematurity (ROP) is increasing, primarily due to advancements in neonatal intensive care that allow more premature infants to survive into infancy. While the majority of premature infants exhibit mild ROP symptoms, only a small percentage advance to severe and potentially sight-threatening stages of the disease. In the United States, approximately 14 thousand preterm infants are estimated to be afflicted by ROP annually, and nearly 90% of these cases experience spontaneous regression of the condition [1]. The most current epidemiological review of screening guidelines and incidence of ROP highlighted significant differences between European countries [2]. It also raises the issue of standardizing screening guidelines in different medical care systems in neonatal units. Furthermore, it highlights the importance of genetic variations that impact differences in the progression of the disease between Eastern and Western Europe, as well as Scandinavian and Balkan countries. Nonetheless, ROP-related visual impairment impacts over 20 thousand infants annually, persisting as a prevalent cause of pediatric visual impairment in the world [3,4].

According to the available data, around 350,000 children were born last year in Poland, of which 7% were born preterm [5]. There is no central registry of ROP patients in our country, so exact statistics on the disease are unknown. Based on the results of our earlier study, approximately 25.9% of screened patients were diagnosed with ROP, and 6.1% of them required treatment for the disease. It seems that the incidence of ROP and ROP requiring treatment rates in Poland is higher than in Western Europe or the USA [6].

Primary prevention of long-term consequences has focused on the identification of the risk factors underlying ROP development. Low gestational age (GA) and birth weight (BW), as well as uncontrolled oxygen supplementation, are known determinants of this condition [3,4]. Additional potential susceptibility risk factors include diminished Apgar scores, inflammation or other complications stemming from premature birth, such as bronchopulmonary dysplasia (BPD), intraventricular hemorrhage (IVH) or necrotizing enterocolitis (NEC). Despite the knowledge of the above relationships, the mechanisms underlying ROP development have not been fully explained [7].

Current scientific reports suggest that the explanation of individual differences in susceptibility to ROP could be sought in the field of genetic background. Thus, many genetic studies on diseases in preterm infants, including ours, focus on key gene variants that enhance the inflammatory [8] or angiogenic [9,10] response and may contribute to an increased risk of preterm birth and complications of prematurity, such as ROP. 

The matrix metalloproteinases (MMPs) are zinc-dependent endopeptidases involved in the modulation of the cellular microenvironment through their impact on the degradation of the extracellular matrix (ECM) [11]. These proteolytic enzymes participate in numerous physiological processes, encompassing embryonic development, the healing of wounds and the process of angiogenesis. To maintain overall homeostasis, MMP activity is regulated by endogenous inhibitors referred to as tissue inhibitors of metalloproteinases (TIMPs) [12]. Maintaining an optimal balance between MMPs and TIMPs is crucial for the proper functionality of healthy tissue. The function of MMPs in angiogenesis involves aiding the invasion and migration of microvascular endothelial cells through the basement membrane of the capillary and into the surrounding ECM. Through ECM degradation, proteinases can also release growth factors, angiogenic factors or angiogenic inhibitors from the cell surface. The interaction between MMPs and TIMPs is essential for angiogenesis, and an improper balance between regulatory mechanisms, coupled with the overexpression of MMPs, can contribute to various pathological conditions, including scarring, inflammatory diseases and tumor angiogenesis [13]. 

Based on current knowledge, elevated MMP activity is linked to retinal neovascularization in numerous ocular conditions, such as age-related macular degeneration (AMD) and diabetic retinopathy [14]; therefore, the role of MMP expression in the pathogenesis of ROP can also be assumed. According to the literature, the level of MMP-1 was found to correlate with VEGF level in the vitreous fluid and modulate angiogenesis in patients with diabetic retinopathy [15]. Further, elevated MMP-1 level was observed in retinal pigment epithelial cells exposed to oxidative stress in the exudative AMD development [16]. Recent studies have demonstrated the link between increased expression of inflammatory markers, including MMP-2 and MMP-9, in amniotic fluid during pregnancy or in tears of premature neonates and ROP development and progression [17,18,19]. However, candidate gene analysis, evaluating the role of 33 single nucleotide variants (SNPs) spanning the whole sequence of *MMP-2* and *MMP-9* genes, found only one association with the occurrence of ROP. The functional basis of this association was not clarified because the identified *MMP-2* rs2285052 risk variant is located within an intronic sequence. Moreover, this variant was relatively rare in the studied Iranian population (frequency 4–9%), so this association does not explain the vast proportion of interindividual differences in susceptibility to ROP. Another study, in patients of Hispanic origin, demonstrated the role of *MMPs* and *TIMPs* genotype of mother or fetus as risk factors for spontaneous preterm labor with intact membranes [20]. This may also suggest their influence on complications of prematurity.

The impact of genetic variance in MMPs and their tissue inhibitors on ROP in Caucasian populations has not yet been analyzed. This study aims to comprehensively investigate the association between variants of *MMP-1*, *MMP-9, TIMP-1* and *TIMP-2* genes and ROP in a Polish cohort of premature infants. Single variant tests of association, haplotype analysis and multi-locus analysis were performed. Demonstrating the role of the variability of these candidate genes in the development and clinical course of ROP may provide new information on the pathogenesis of this disease.

## 2. Results

### 2.1. Clinical Data

The demographic and clinical features of the patients are presented in Table 1. A total of 100 patients (47 female) were enrolled in the study, with a median GA of 28 weeks (range 22–33 weeks) and a median BW of 1080 g (range 432–2010 g). ROP was diagnosed in 50 patients (92 eyes; in eight patients, the disease was diagnosed in a single eye), including 28 patients (49 eyes) with spontaneous regression of the disease and 22 patients (43 eyes) with ROP requiring treatment. Patients were treated with laser photocoagulation (n = 14; 27 eyes), anti-vascular endothelial growth factor (anti-VEGF) therapy (n = 6; 12 eyes) or both methods (n = 2; 4 eyes). 

As shown in Table 1, a relationship between low GA, BW, Apgar score; prolonged mechanical ventilation; the presence of IVH or BPD and ROP development was observed. Low GA, BW, prolonged mechanical ventilation, and the presence of IVH were also associated with the progression of ROP to advanced stages.

### 2.2. Association Studies 

#### 2.2.1. Single Variant Tests of Association

Table 2 and Table 3 report the frequencies of *MMPs* and *TIMPs* alleles and genotypes, comparing patients with and without ROP. Genotype distribution for the tested variants showed no deviation from the Hardy–Weinberg equilibrium (HWE) in both groups (*p* < 0.05; Table 2), except for the rs4898 *TIMP*-1 variant in the whole group, which is located on the X chromosome. In female infants with all three rs4898 genotypes, the allelic distribution was consistent with HWE (*p* = 0.065). 

The allele frequency in newborns with ROP did not differ significantly from that in newborns without ROP, although non-significant trends were observed for the three variants. The *MMP-1* rs1799750 1G deletion allele occurred at a higher frequency in ROP cases compared to no-ROP cases (0.50 vs. 0.39; odds ratio (OR) = 1.56; *p* = 0.117; Table 2). Similarly, the frequency of the *TIMP-2* rs2277698T allele was higher in cases of ROP than in infants without ROP (0.14 vs. 0.08; OR = 1.87; *p* = 0.175). In contrast, for the *TIMP-1* polymorphism, the frequency of the rs4898C allele was lower in female ROP cases compared to no-ROP controls (OR = 0.49; *p* = 0.087).

The association between ROP and studied variants was analyzed using logistic regression. Crude ORs and adjusted (AORs) for GA and BW were computed. Since *TIMP-1* is an X-linked gene, the analysis for rs4898 was performed in males and females separately. The codominant genetic model was tested as the main model; in addition, results were obtained for dominant, recessive, overdominant and log-additive models (Table 3).

Association of the *MMP-1* rs1799750 (whole group) and *TIMP-1* rs4898 (female newborns) variants with ROP incidence was found, while no association with ROP was observed for the *MMP-9* and *TIMP-2* gene variants studied. We further investigated gene-environment interactions between *MMP-1* and *TIMP-1* genotypes and factors such as GA, BW and sex. We found that *MMP-1* rs1799750 1G allele in the entire group and *TIMP-1* rs4898 C allele in female newborns can interact with GA < 28 weeks in a multiplicative manner, increasing or decreasing the risk of ROP (refer to the Supplemental data Table S1 for details).

The relationship between the studied variants and ROP advancement was examined in the stratified analysis comparing patients with spontaneous ROP regression and those with ROP requiring treatment and an additional comparison of the control group (without ROP) with the subgroup of patients that developed the most severe ROP requiring treatment. In female infants, the frequency of *TIMP-1* rs4898 genotypes was statistically significantly different between the groups no-ROP (n = 25) and ROP requiring treatment (n = 10; *p* = 0.043 in the codominant model). The genotype frequency distribution for TT homozygotes, TC heterozygotes and CC homozygotes was 12.0%, 68.0% and 20.0%, respectively, in children without ROP and 50.0%, 50.0% and 0.0% in children with ROP requiring treatment. 

The conducted analysis of the association of genotypes and alleles of the studied genetic variants with newborn clinical data did not show any statistically significant relationship. 

#### 2.2.2. Haplotype Analysis

**Figure 1:**
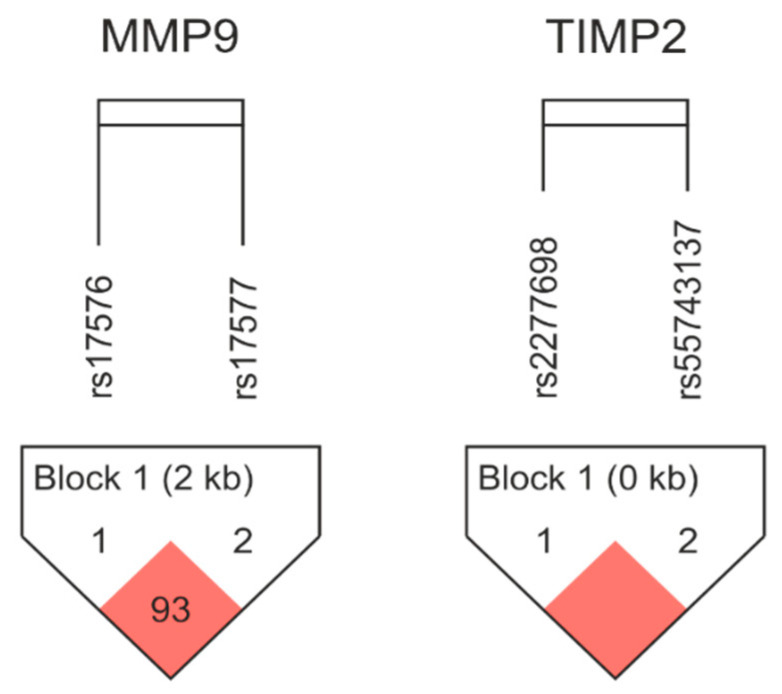
Linkage disequilibrium (LD) plots containing two SNPs from *MMP-9* and two SNPs from *TIMP-2*. Red squares display statistically significant associations between a pair of SNPs, as measured by Lewontin’s *D*′ determined by Haploview software.

An analysis of linkage disequilibrium (LD) and haplotype frequencies of the studied variants of the *MMP-9* and *TIMP-2* genes was performed using Haploview 4.2 (http://www.broad.mit.edu/mpg/haploview/ accessed on 10 January 2023). A strong LD was found between the analyzed variants (for *MMP-9* rs17576/rs17577 distance 2886 bp, *D*’ = 0.938, *r*^2^ = 0.3 and for *TIMP-2* rs2277698/rs55743137 distance 168 bp, *D*’ = 1.0, *r*^2^ = 0.7). A schematic diagram of the LD pattern is shown in Figure 1. 

The frequencies of haplotypes in the studied groups of newborns were analyzed; the results are presented in Table 4.

The strongest relationship was observed for the *MMP-9* GG haplotype (rs17576/rs17577), which was more common in the ROP group (0.251 vs. 0.151 in no-ROP, χ^2^ = 3.103, *p* = 0.078). An in silico analysis of the frequency of occurrence of haplotypes analyzed in the work of polymorphic variants in European populations from the 1000Genomes project was also carried out using LDLink (https://ldlink.nih.gov/, accessed on 31 May 2023) [21]. The results for these populations were comparable to ours. For *MMP-9* rs17576/rs17577 *D*’ = 0.991, *r*^2^ = 0.3, haplotypes they were AG = 0.618, GG = 0.207, GA = 0.174 and AA = 0.001, whereas for *TIMP-2* rs2277698/rs55743137 *D*’ = 1.0, *r*^2^ = 0.6, haplotypes, they were CT = 0.800, TG = 0.125, CG = 0.075 and TT = 0.000.

#### 2.2.3. Gene-Gene Interaction Analysis

**Figure 2:**
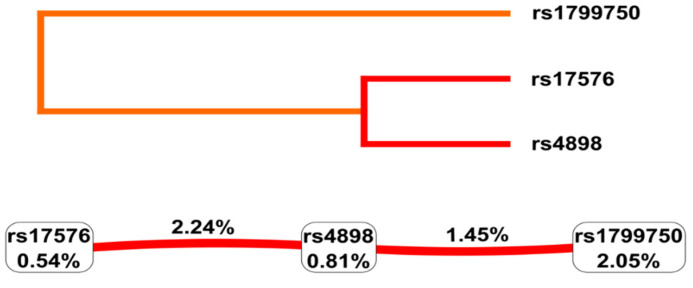
The dendrogram (top) and Fruchterman Rheingold (bottom) plots obtained by multifactor dimensionality reduction SNP-SNP interaction for the risk of retinopathy of prematurity. The red line colors denote strong and orange moderate synergism.

The influence of SNP-SNP interactions on the predisposition to ROP was examined by multifactor dimensionality reduction (MDR 3.0.2), as summarized in Table 5 and Figure 2. The best one-locus model was *MMP-1* rs1799750 (testing accuracy 44%; CVC 6/10; *p* = 0.096). But the best interaction model was a three-loci model (*MMP-1* rs1799750, *MMP-9* rs17576, *TIMP-1* rs4989) with testing accuracy to 42% (CVC 9/10; *p* = 0.0003).

Analysis of the ROP and no-ROP controls dataset revealed synergistic interactions between *TIMP-1* rs4898–*MMP-9* rs17576 (IG = 2.24%) and also between *MMP-1* rs1799750–*TIMP-1* rs4898 (IG = 1.45%; Figure 2).

## 3. Discussion

Currently, prematurity is considered to be a worldwide concern, with an incidence of 15 million preterm infants each year [22]. One of the diseases affecting preterm infants is ROP. It is a disorder of immature retinal vasculature that can result in serious complications such as retinal detachment and blindness. The incidence of ROP differs between European countries, which in part can be explained by genetic background [2].

The main risk factors for ROP, such as GA and BW, were confirmed in our studies to be inversely correlated with the development of the disease [3,4]. Moreover, we found statistically significant associations between the development of ROP and lower median Apgar score in the first and fifth minute after birth, prolonged mechanical ventilation and the occurrence of IVH and BPD, which were also suggested in other studies [7]. Our further analysis showed that the duration of mechanical ventilation and the occurrence of IVH were factors that contributed to the development of more severe ROP requiring treatment. However, the pathogenesis of the disease is not fully understood, and it is uncertain why some patients experience spontaneous regression while others require treatment. The identification of genetic factors predisposing to ROP could explain the underlying mechanisms of the disease and potentially predict treatment outcomes based on an individual’s genotype.

In the present study, we investigated the associations of variants in genes encoding *MMP-1*, *MMP-9*, *TIMP-1* and *TIMP-2* with ROP in a Polish cohort of premature infants. The studied variants were previously found to have functional consequences. The *MMP-1* rs179975 variant is an insertion/deletion polymorphism of a single guanine (2G or 1G) located at nucleotide 1607 in the *MMP-1* gene promoter. The presence of the additional guanine (2G allele) was previously associated with increased transcriptional activity, modifying the level of *MMP-1* expression [23,24]. *MMP-9* rs17576 variant is located in the gelatinase-specific fibronectin type II domains, which may enhance substrate binding, whereas rs17577 is located in the hemopexin domain, which is considered to influence both substrate and inhibitor binding [25]. Both selected variants of the *MMP-9* gene are located in the coding sequence and change the amino acid sequence of the protein (Gln279Arg and Arg668Gln, respectively). *TIMP-1* rs4898 is an exon 5 variant localized on chromosome X; therefore, men are monoallelic T or C, whereas women can be TT and CC homozygotes and TC heterozygotes. T allele in the 372 T>C polymorphism of *TIMP-1* is associated with increased production of *TIMP-1* [18]. The rs2277698 variant of the *TIMP-2* gene is located in exon 3 and changes cytosine to thymine but does not affect the protein sequence (Ser101=). The second of the studied *TIMP-2* polymorphisms (rs55743137) is located in intron 2 and causes the replacement of thymine with guanine. We propose that demonstrating the importance of these variants for ROP may lead to a better understanding of the pathogenesis of this disease. 

In our study, we found a statistically significant positive association between the *MMP-1* rs179975 1G deletion allele and ROP in the log-additive model (adjusted OR = 5.01, *p* = 0.048). Moreover, in female neonates, the negative association between the *TIMP-1* rs4898C allele and the occurrence of ROP and ROP requiring treatment was observed (both in the codominant model, with respective *p*-values < 0.05 and 0.043). The strongest effect was observed for CC homozygotes, the frequency of which in the group with ROP was 4.5%, compared to 20% in the subgroup without ROP. The effects of these variants may modify the effect of low gestational age on the risk of ROP. There were no statistically significant associations between single variants or haplotypes of the *MMP-9* or *TIMP-2* gene and ROP; however, two and three loci interactions between *MMP-1* rs1799750 and *TIMP-1* rs4989 (*p* = 0.015), as well as *MMP-1* rs1799750, *MMP-9* rs17576 and *TIMP-1* rs4989 (*p* = 0.0003), influencing the risk of ROP were observed. Several minor findings were also identified. We observed a strong LD between studied *MMP-9* rs17576/rs17577 and *TIMP-2* rs2277698/rs55743137 variants in our population, which, according to the results of our in silico analyses, conducted on data from the 1000Genomes project, was similar to other European populations. Moreover, the analysis of haplotype frequencies revealed the slightly increased frequency of the *MMP-9* GG haplotype in cases with ROP.

MMPs constitute a family of proteolytic endopeptidases whose activity is controlled by their tissue inhibitors. By regulating a wide range of biological processes, these enzymes are responsible for the maintenance and remodeling of various tissues, as well as eye structures. To the best of our knowledge, the involvement of the specific MMPs and TIMPs in ocular diseases has already been proposed, including macular degeneration, glaucoma and diabetic retinopathy [14]. According to the literature, the altered expression of MMP and TIMP genes has been suggested as a risk factor contributing to preterm birth. The influence of variations in genes encoding for these proteins in both mother and fetus on preterm labor was for the first time demonstrated in 2010 by Romero et al. [20]. However, the results of subsequent studies are not consistent. Studies conducted by Padney et al. confirmed that the *MMP-9* gene played a significant role in preterm labor. An increased risk was found in mothers homozygous for the promoter variant (rs3918242; −1562 C>T [26]. The mechanism of preterm birth was interpreted by authors as excessive degradation of the amniochorionic ECM, leading to rupture of the membranes. On the other hand, no evident associations were found in this study for the *MMP-1* rs1799750 1G/2G promoter variant, which was confirmed by Pereza et al. in another study [24]. On the contrary, Fujimoto et al. explored the same variant and found a significant association between fetal carriage of the 2G allele and preterm premature rupture of membranes [27]. In another study, Lathouras et al. reported that different polymorphisms of *MMP-1*, *-3* and *TIMP-2* were not associated with premature labor [28]. 

Metalloproteinases are also considered to act as mediators of inflammatory processes by interacting with specific extracellular targets, such as receptors, cytokines, growth factors and adhesion molecules [14]. Specifically, it was suggested that the regulatory function of MMPs had an important role in the pathogenesis of retinal vascular diseases. Patnaik et al. reported that preterm infants with ROP had increased activity of MMPs in the vitreous and tears, which was correlated with the severity of the disease [29]. Further, they presented that MMP-9 regulated the expression of opticin under hypoxic stress. Results from both mouse and rat models of oxygen-induced retinopathy suggest that MMP-2 plays a dominant role in retinal angiogenesis and that MMP-2 inhibition may be a viable therapeutic approach in ocular diseases characterized by retinal neovascularization [30]. MMPs play a vital role in angiogenesis by facilitating the degradation of the capillary basement membrane and enabling the invasion of activated endothelial cells into surrounding tissues. Additionally, a significant inhibition of neovascularization when an MMP inhibitor was administered intraperitoneally was noted, suggesting the potential consideration of this mechanism as an alternative therapy for ROP. In another study, Lorente et al. showed that septic patients with the T allele of the *TIMP-1* rs4898 variant had higher TIMP-1 serum levels and a lower survival rate. A relationship between the inflammation response in sepsis and MMP/TIMP balance was suggested [31]. The frequency of this allele was increased in female ROP cases, which suggested the role of sepsis and inflammation in the pathogenesis of ROP. These findings are in line with our previous observation of the involvement of genetic variations in the *SELENOP* gene, an important factor for the suppression of the antioxidant barrier and the immune system, in the pathogenesis of the disease [8]. Among other factors that may modulate the influence of the studied genetic variants, hypoxia at birth can be mentioned, which has been identified as a factor that increases the risk of ROP fourfold [9]. In this study, we also observed that birth asphyxia may be a risk factor for ROP and its progression to advanced stages. Observed frequencies were 6% in non-ROP patients vs. 14% in ROP cases and 22.7% in treatment-requiring ROP; however, the differences were not statistically significant.

The main limitation of the current study is the relatively small sample size. Therefore, the current findings will need to be tested with a larger data set. The lack of statistical power in the study may have also contributed to the inability to establish the significance of single variants for the *MMP-9* and *TIMP-2* genes that were investigated. Nevertheless, the strength of this work arises from the homogenous population of Caucasian neonates. This presents importance for future use of our data in meta-analysis studies.

## 4. Materials and Methods

The study was conducted in accordance with the Declaration of Helsinki and approved by the Bioethics Committee of Poznan University of Medical Sciences (45/22 and 126/22, both for patients from control and case groups). 

### 4.1. Study Population

This prospective study was conducted at the Clinical Hospital of Gynecology and Obstetrics, Poznan University of Medical Sciences in Poznan, Poland, from 1 March 2014 to 14 January 2020. A population of 100 consecutive preterm infants born alive was selected for the study. The inclusion criteria were (1) preterm birth between 22 + 0 and 33 + 0 weeks of GA and (2) written consent from the child’s parents/guardians for inclusion in the study. Based on the results of the retinal screening examinations, this population was divided into cases (preterm infants diagnosed with ROP; n = 50) and controls (preterm infants without ROP; n = 50). The group of patients who developed ROP was later categorized into subgroups: those with spontaneous ROP regression (n = 28) and those requiring treatment for ROP (n = 22). All patients and their parents were Caucasian.

The following criteria were determined for exclusion from the control and case groups: chromosomal abnormalities, multiple pregnancies, pregnancies involving death of one of the fetuses, death before reaching 40 weeks of postmenstrual age and diagnoses of inherited metabolic disorders. Patients diagnosed with congenital toxoplasmosis, rubella, cytomegalovirus, herpes or others (TORCH) were also excluded from the study due to possible retinal involvement.

### 4.2. Clinical Features

The clinical features that might be related to ROP development were recorded, including gender, BW (grams), GA (weeks), mode of delivery, Apgar score at the 1st and 5th minute, duration of ventilation support (days), birth asphyxia (determined as Apgar score less than 6 at the 10th minute and pH < 7.0 or blood base excess [BE] < −15 mmol/L in cord blood), intrauterine infection (confirmed by a positive culture in originally sterile samples combined with clinical symptoms or the development of pneumonia within the initial 48 h after birth), late-onset infection (comprising pneumonia, sepsis or urinary tract infections) and prematurity-related complications such as BPD, IVH and NEC.

### 4.3. ROP Diagnosis

In line with the consensus among Polish Neonatologists and the Pediatric Ophthalmology Section concerning ROP screening, all infants enrolled in the study that were born ≤ 33 weeks of GA with BW ≤ 1800 g or those who did not fulfill these criteria but were qualified by the neonatologist as at high risk of ROP, underwent subsequent ophthalmological examinations. The first eye fundus examination was performed in the 4th week after birth, and further examinations, depending on retinal vascularization, were carried out every 7–10 days. The decision to finish examinations was made in patients with vascularized zone III of the retina or ROP regression observed on at least two consecutive visits. In the case of ROP diagnosis, fundus lesions were reported in accordance with the International Classification of Retinopathy of Prematurity (ICROP). The stage, retinal zone, presence of plus disease and state of retinal vessels were determined [32].

### 4.4. ROP Treatment

Patients who developed ROP were systematically examined; in some cases, the disease resolved spontaneously, but others developed ROP requiring treatment. In accordance with the Early Treatment for Retinopathy of Prematurity guidelines, patients received treatment within 72 h after the diagnosis, including any of the following: stage of ROP in Zone I with plus disease, stage 3 ROP without plus disease in Zone I or stage 2 or 3 ROP with plus disease in Zone II [33]. Due to the lack of strict indications for treatment methods in the global guidelines, laser treatment remained the method of choice for ROP treatment. However, intravitreal injection of anti-VEGF antibody (ranibizumab) was used in patients who developed severe zone I ROP. In some cases, anti-VEGF therapy was used in combination with laser photocoagulation.

### 4.5. Studied Genetic Variants

In our study, candidate genes were chosen based on their potential role in the development of ROP. Single nucleotide variants were selected from the SNP database (dbSNP) of the National Center for Biotechnology Information (NCBI) (http://www.ncbi.nlm.nih.gov/projects/SNP, accessed on 10 January 2023). We specifically focused on variants with a minor allele frequency (MAF) of at least 5% in European populations. We studied six variants: *MMP-1* rs179975, *MMP-9* rs17576, *MMP-9* rs17577, *TIMP-1* rs4898, *TIMP-2* rs2277698 and *TIMP-2* rs55743137. Details regarding these variants are presented in Table 6.

Peripheral venous blood samples (0.5 mL) were collected after the delivery and stored. Genomic DNA extraction was performed with the QIAamp DNA Blood Mini Kit (QIAGEN Inc.; Hilden, Germany) according to the manufacturer’s instructions. The studied polymorphisms were genotyped through polymerase chain reaction (PCR) and restriction fragment length polymorphism (RFLP) techniques. The primers and restriction enzymes (Thermo Fisher Scientific; Waltham, MA, USA) employed in these reactions were sourced from previously published studies and are detailed in Table 7 [23,25,28,31,34]. Subsequently, DNA fragments were examined by electrophoresis on agarose gels using Midori Green Advance DNA Stain (Nippon Genetics, Düren, Germany). For quality control, blind repeats of approximately 5% of samples were included. All variants had a call rate greater than 95%.

### 4.6. Statistical Analysis

The results were presented as either frequencies and percentages for categorical variables or as medians and interquartile ranges for continuous variables that did not follow a normal distribution. The normality of variable distribution was tested using the Shapiro–Wilk test. To determine the relationship between categorical variables and ROP, the following tests were performed: the Fisher exact probability test, the *χ*^2^ test, the Fisher Freeman Halton test and the *χ*^2^ test with Yates correction. For non-normally distributed continuous variables, the Mann–Whitney test was used to assess differences. The associations between studied genetic variants and ROP were assessed using the odds ratio (OR) in univariate analyses and the adjusted odds ratio (AOR) in multivariate analyses after adjustment for GA and BW. The 95% confidence interval (95% CI) was computed using logistic regression. Variants were assessed for Hardy–Weinberg equilibrium (HWE). To examine the relationships between each variant and ROP, multiple inheritance models (including codominant, dominant, recessive, overdominant and log-additive) were selected. The optimal inheritance models were chosen based on the Akaike information criterion (AIC). Statistical analysis was conducted using R software version 4.1.2 (R Foundation for Statistical Computing, Vienna, Austria, accessed on 22 May 2023) [35] and the SNPassoc package [36]. LD among the selected variants was calculated using Haploview v.4.2 software [37]. Interaction analyses were performed using the open-source MDR v.3.0.2 software [38]. Gene-environment interaction analysis was performed using the method described by Botto and Khoury [39]. Statistically significant results were indicated by *p*-values less than 0.05.

## 5. Conclusions

In conclusion, our findings suggest that *MMP-1* rs179975 and *TIMP-1* rs4898 variants, as well as interactions between genes encoding MMPs and TIMPs, may impact the development of ROP in a Polish cohort of premature infants. However, no statistically significant evidence was found confirming the individual role of *MMP-9* rs17576, rs17577 and *TIMP-2* rs2277698, rs55743137 in the development of this disease. 

## Figures and Tables

**Table 1:** Characteristics of patients.

Characteristic	I No-ROP (n = 50)	II ROP (n = 50)	II vs. I *p*-Value (Person)	IIa ROP with Regression (n = 28)	IIb ROP with Treatment (n = 22)	IIb vs. IIa *p*-Value (Person)
Sex, n (%)			0.548 ^a^			0.918 ^b^
Female	25 (50.0)	22 (44.0)		12 (42.9)	10 (45.5)	
Male	25 (50.0)	28 (56.0)		16 (57.1)	12 (54.5)	
Gestational age (weeks), median (range)	30 (26–33)	26 (22–31)	<0.0001 ^d^	28 (23–31)	25 (22–30)	0.005 ^d^
Birth weight (grams)	1350	855	<0.0001 ^c^	948	755	0.014 ^c^
median (range)	(790–2010)	(432–1500)		(610–1500)	(432–1485)	
Apgar score, median (range)			<0.0001 ^d^ 0.0003 ^d^			0.547 ^d^ 0.299 ^d^
1st minute	6 (1–10)	4 (1–9)		5 (1–9)	4 (1–6)	
5th minute	8 (5–10)	7 (1–10)		7 (1–9)	7 (4–10)	
Mode of delivery, n (%)	18 (32.0) 32 (64.0)		0.159 ^a^			0.828 ^b^
Vaginal		23 (46.0)		12 (42.9)	11 (50.0)	
Caesarean section		27 (54.0)		16 (57.1)	11 (50.0)	
Birth asphyxia, n (%)	3 (6.0)	7 (14.0)	0.317 ^b^	2 (7.1)	5 (22.7)	0.245 ^b^
Mechanical ventilation (days) median (range)	9 (1–53)	55.5 (3–146)	<0.0001 ^d^	45 (3–101)	80 (3–146)	0.0001 ^c^
Intrauterine infection, n (%)	25 (50.0)	33 (66.0)	0.105 ^a^	17 (60.7)	16 (72.7)	0.556 ^b^
Late-onset infection, n (%)	6 (12.0)	13 (26.0)	0.126 ^b^	6 (21.4)	7 (31.8)	0.612 ^b^
IVH, n (%)	9 (18.0)	12 (24.0)	0.0003 ^a^	4 (14.3)	8 (36.4)	0.017 ^b^
BPD, n (%)	7 (14.0)	31 (62.0)	<0.0001 ^a^	14 (50.0)	17 (77.3)	0.093 ^b^
NEC, n (%)	15 (30.0)	33 (66.0)	0.461 ^a^	14 (50.0)	19 (86.4)	0.139 ^b^

Abbreviations are as follows: bronchopulmonary dysplasia (BPD), intraventricular hemorrhage (IVH), necrotizing enterocolitis (NEC); statistical analysis: a—*χ*^2^ test, b—*χ*^2^ test with Yate’s correction, c—*t*-student test, d—Mann–Whitney test.

**Table 2:** Distribution of studied variants in ROP and no-ROP subjects with the analysis of differences in allele frequency.

Gene and Variant	I No-ROP n (%)	II ROP n (%)	Comparison of Groups II vs. I	
			OR (95%CI)	*p*-Value (Person)
*MMP-1* rs1799750			1.56 (0.89–2.74)	0.117
2G	61 (0.61)	50 (0.50)		
1G	39 (0.39)	50 (0.50)		
HWE *p*-value (Person)	0.814	0.157		
*MMP-9* rs17576			1.30 (0.73–2.33)	0.374
A	68 (0.68)	62 (0.62)		
G	32 (0.32)	38 (0.38)		
HWE *p*-value (Person)	0.567	0.640		
*MMP-9* rs17577			0.68 (0.313–1.48)	0.328
G	82 (0.82)	87 (0.87)		
A	18 (0.18)	13 (0.13)		
HWE *p*-value (Person)	0.716	0.291		
*TIMP-1* rs4898; All infants			0.76 (0.43–1.32)	0.323
T	47 (0.47)	54 (0.54)		
C	53 (0.53)	46 (0.46)		
HWE *p*-value (Person)	0.025	0.002		
*TIMP-1* rs4898; Female			0.49 (0.21–1.12)	0.087
T	23 (0.46)	28 (0.64)		
C	27 (0.54)	16 (0.36)		
HWE *p*-value (Person)	0.065	0.079		
*TIMP-1* rs4898; Male			1.07 (0.36–3.14)	0.909
T	12 (0.48)	13 (0.46)		
C	13 (0.52)	15 (0.54)		
*TIMP-2* rs2277698			1.87 (0.75–4.68)	0.175
C	92 (0.92)	86 (0.86)		
T	8 (0.08)	14 (0.14)		
HWE *p*-value (Person)	0.539	0.231		
*TIMP-2* rs55743137			1.37 (0.626–3.00)	0.428
T	87 (0.87)	83 (0.83)		
G	13 (0.13)	17 (0.17)		
HWE *p*-value (Person)	0.846	0.578		

Abbreviations: HWE—Hardy–Weinberg equilibrium.

**Table 3:** Genotype distribution in the studied infants and analysis of the association between individual variants of *MMP-1*, *MMP-9*, *TIMP-1* and *TIMP-2* genes and the occurrence of ROP. Statistically significant results are given in bold font.

Gene, SNP	Genotypes and Tested Models	I no-ROP n (%)	II ROP n (%)	II vs. I Co-Dominant Model					
				Crude			Adjusted		
				OR (95%CI)	*p*	AIC	AOR (95%CI)	*p*	AIC
*MMP-1* rs1799750	*2G/2G*	19 (38.0)	15 (30.0)	1.00	0.241	141.8	1.00	0.065	83.5
	*2G/1G*	23 (46.0)	20 (40.0)	1.10 (0.45–2.72)			1.55 (0.38–6.30)		
	*1G/1G*	8 (16.0)	15 (30.0)	2.38 (0.80–7.09)			6.38 (0.84–48.4)		
	Dominant	31 (62.0)	35 (70.0)	1.43 (0.62–3.29)	0.398	141.9	2.25 (0.61–8.34)	0.219	81.8
	Recessive	8 (16.0)	15 (30.0)	2.25 (0.85–5.93)	0.096101	139.8	4.14 (0.93–18.33)	0.061	84.3
	Overdominant	27 (54.0)	30 (60.0)	1.28 (0.58–2.82)	0.544	142.3	1.33 (0.41–4.27)	0.631	85.7
	Log-additive	50 (50.0)	50 (50.0)	2.22 (0.75–6.58)	0.144	140.4	**5.01 (1.03–28.4)**	**0.048**	**82.0**
*MMP-9* rs17576	*AA*	24 (48.0)	20 (40.0)	1.00	0.689	143.9	1.00	0.454	86.4
	*AG*	20 (40.0)	22 (44.0)	1.32 (0.57–3.08)			1.92 (0.57–6.40)		
	*GG*	6 (12.0)	8 (16.0)	1.60 (0.48–5.38)			2.50 (0.38–16.45)		
	Dominant	26 (52.0)	30 (60.0)	1.38 (0.63–3.06)	0.420	142.0	2.02 (0.65–6.34)	0.220	84.4
	Recessive	6 (12.0)	8 (16.0)	1.40 (0.45–4.37)	0.564	142.3	1.83 (0.31–10.77)	0.506	85.5
	Overdominant	30 (60.0)	28 (56.0)	1.18 (0.53–2.61)	0.685	142.5	1.60 (0.51–4.99)	0.415	85.3
	Log-additive	50 (50.0)	50 (50.0)	1.28 (0.73–2.25)	0.391	141.9	1.68 (0.72–3.92)	0.222	84.4
*MMP-9* rs17577	*GG*	34 (68.0)	37 (74.0)	1.00	0.495	141.7	1.00	0.690	87.2
	*GA*	14 (28.0)	13 (26.0)	0.85 (0.35–2.07)			0.85 (0.22–3.21)		
	*AA*	2 (4.0)	0 (0.0)	―			―		
	Dominant	16 (32.0)	13 (26.0)	0.75 (0.31–1.78)	0.508	142.2	0.77 (0.21–2.83)	0.692	85.8
	Recessive	2 (4.0)	0 (0.0)	―	0.495	139.8	―	0.409	85.2
	Overdominant	36 (72.0)	37 (74.0)	0.90 (0.37–2.19)	0.822	142.6	0.87 (0.23–3.31)	0.835	85.9
	Log-additive	50 (50.0)	50 (50.0)	0.67 (0.30–1.48)	0.495	141.6	0.72 (0.22–2.42)	0.594	85.6
*TIMP-1* rs4898	Female newborns								
	*TT*	3 (12.0)	7 (31.8)	1.00	0.098	66.3	**1.00**	**<0.050**	**44.8**
	*TC*	17 (68.0)	14 (63.6)	0.35 (0.08–1.62)			**0.10 (0.01–1.13)**		
	*CC*	5 (20.0)	1 (4.5)	0.09 (0.01–1.08)			**0.02 (0.01–0.81)**		
	Dominant	22 (88.0)	15 (68.2)	0.29 (0.06–1.31)	0.154	66.0	**0.09 (0.01–1.04)**	**0.048**	**43.1**
	Recessive	5 (20.0)	1 (4.5)	0.19 (0.02–1.78)	0.194	65.9	0.36 (0.01–11.3)	0.554	43.4
	Overdominant	8 (32.0)	8 (36.3)	1.21 (0.36–4.07)	0.768	66.1	1.50 (0.49–4.59)	0.515	43.3
	Log-additive	50 (50.0)	50 (50.0)	**0.09 (0.01–1.04)**	**0.047**	61.7	0.02 (0.01–1.07)	0.050	44.6
	Male newborns								
	*T*	12 (48.0)	13 (46.4)	1.00	0.909	77.3	1.00	0.189	34.9
	*C*	13 (52.0)	15 (53.6)	1.07 (0.36–3.14)			0.25 (0.03–1.99)		
*TIMP-2* rs2277698	*CC*	42 (84.0)	38 (76.0)	1.00	0.397	141.4	1.00	0.249	85.2
	*CT*	8 (16.0)	10 (20.0)	1.38 (0.49–3.86)			1.15 (0.26–5.08)		
	*TT*	0 (0.0)	2 (4.0)	―			―		
	Dominant	8 (16.0)	12 (24.0)	1.66 (0.61–4.49)	0.316	141.6	1.63 (0.41–6.54)	0.490	85.5
	Recessive	0 (0.0)	2 (4.0)	―	0.495	139.8	―	0.098	83.2
	Overdominant	42 (84.0)	40 (80.0)	1.31 (0.47–3.66)	0.602	142.4	1.01 (0.23–4.42)	0.993	85.9
	Log-additive	50 (50.0)	50 (50.0)	1.80 (0.73–4.41)	0.397	140.9	1.89 (0.59–6.08)	0.272	84.7
*TIMP-2* rs55743137	*TT*	38 (76.0)	35 (70.0)	1.00	0.730	144.0	1.00	0.452	86.3
	*TG*	11 (22.0)	13 (26.0)	1.28 (0.51–3.24)			1.12 (0.30–4.14)		
	*GG*	1 (2.0)	2 (4.0)	2.17 (0.19–25.01)			6.32 (0.31–5130.78)		
	Dominant	12 (24.0)	15 (30.0)	1.36 (0.56–3.30)	0.499	142.2	1.43 (0.42–4.88)	0.571	85.6
	Recessive	1 (2.0)	2 (4.0)	2.04 (0.18–23.27)	0.554	142.3	6.15 (0.30–125.33)	0.211	84.4
	Overdominant	39 (78.0)	37 (74.0)	1.25 (0.50–3.13)	0.639	142.4	1.00 (0.27–3.65)	0.998	85.9
	Log-additive	50 (50.0)	50 (50.0)	1.35 (0.63–2.89)	0.440	142.0	1.60 (0.58–4.40)	0.358	85.1

**Table 4:** Haplotype analysis of *MMP-9* and *TIMP-2* variants in ROP and no-ROP infants.

Gene and SNPs	Haplotypes	Frequency Overall	Frequency ROP, No-ROP	*p*-Value (Person)
*MMP-9* rs17576/rs17577	AG	0.644	0.619, 0.669	0.462
	GG	0.201	0.251, 0.151	0.078
	GA	0.149	0.129, 0.169	0.428
*TIMP-2* rs2277698/rs55743137	CT	0.850	0.830, 0.870	0.428
	TG	0.110	0.140, 0.080	0.175
	CG	0.040	0.030, 0.050	0.471

**Table 5:** MDR analysis of SNP-SNP interactions in relation to ROP risk.

Loci Model	Gene and Variant	Training Balance Accuracy (%)	Testing Balance Accuracy (%)	CVC	Person *p*-Value
One locus	*MMP-1* rs1799750	57.4	44.0	6/10	0.0962
Two loci	*MMP-1* rs1799750, *TIMP-1* rs4989	63.4	42.1	6/10	0.0150
Three loci	*MMP-1* rs1799750, *MMP-9* rs17576, *TIMP-1* rs4989	68.9	42.3	9/10	0.0003

Abbreviations: CVC—cross-validation consistency.

**Table 6:** Characteristics of selected genes and polymorphisms.

Gene	rs Number	Position (GRCh38.p14)	Allele	Variant Type	MAF
*MMP-1*	rs1799750	chr11:102799765-102799766	delG	Promoter	delG = 0.4960
*MMP-9*	rs17576	chr20:46011586	A>G	Coding Gln279Arg	G = 0.3807
*MMP-9*	rs17577	chr20:46014472	G>A	Coding Arg668Gln	A = 0.1750
*TIMP-1*	rs4898	chrX:47585586	T>C	Coding Phe124=	C = 0.4650
*TIMP-2*	rs2277698	chr17:78870935	C>T	Coding Ser101=	T = 0.1252
*TIMP-2*	rs55743137	chr17:78871103	G>T	Intronic	G = 0.1998

Abbreviations: GRCh38.p14—Genome Reference Consortium Human Build 38 patch release 14, MAF—minor allele frequency based on data from 1000 Genomes Project for European population.

**Table 7:** Primers and PCR-RFLP conditions for studied genetic variants (sequence of primers according to [21,23,25,28,31]).

Gene and Variant	Sequence of Primers	Temperature of Primer Attachment	Restriction Enzyme	PCR Products
*MMP-1* rs179975	5′-TGA CTT TTA AAA CAT AGT CTA TGT TCA 3′ 5′ -TCT TGG ATT GAT TTG AGA TAA GTC ATAGC-3′	50 °C	*AluI*	1G 241, 28 pz 2G 269 pz
*MMP-9* rs17576	5′-GAGAGATGGGATGAACTG-3′ 5′-GTGGTGGAAATGTGGTGT-3′	60 °C	*MspI (HpaII)*	A 252, 187 pz G 187, 129, 123 pz
*MMP-9* rs17577	5′-ACA CGC ACG ACG TCT TCC AGT ATC-3′ 5′-GGG GCA TTT GTT TCC ATT TCC A-3′	63 °C	*TaqI*	G 115, 23 pz A 138 pz
*TIMP-1* rs4898	5’-GCA CAT CAC TAC CTG CAG TCT-3’ 5’-GAA ACA AGC CCA CGA TTT AG-3’	54 °C	*BauI* *(BssI)*	T 175 pz C 153,22 pz
*TIMP-2* rs2277698	5′-CCA GGA AAT TGG CAG GTA GT-3′ 5′-GAA TTC ACC AAC TGT GTG GC-3′	60 °C	*BsrI*	C 369 pz T 231, 138 pz
*TIMP-2* rs55743137	5′-CCT TTG AAC ATC TGG AAA GAC AA-3′ 5′-TAA CCC ATG TAT TTG CAC TTC CT-3′	58 °C	*AluI*	T 160 pz G 108, 52 pz

## Data Availability

The data that support the findings of this study are available from the corresponding author upon reasonable request.

## Supplementary Materials

The following supporting information can be downloaded at: https://www.mdpi.com/article/10.3390/ijms242015309/s1.
