# Supplementary material for: Comprehensive Analysis of the Role of Gene Variants in Matrix Metalloproteinases and Their Tissue Inhibitors in Retinopathy of Prematurity: A Study in the Polish Population
Source: Int J Mol Sci. 2023 Oct 18;24(20):15309. doi: 10.3390/ijms242015309 (PMC10607760; doi:10.3390/ijms242015309)
Supplement: Supplementary file 1 [file ijms-24-15309-s001.zip › ijms-2617807-supplementary.pdf]

Article

Comprehensive Analysis of the Role of Gene Variants in Matrix Metalloproteinases and Their Tissue Inhibitors in Retinopathy of Prematurity: A Study in the Polish Population

Aneta Choręziak-Michalak [1], Dawid Szpecht [2], Anna Chmielarz-Czarnocińska [1], Agnieszka Seremak-Mrozikiewicz [3], Krzysztof Drews [3], Grażyna Kurzawińska [3], Ewa Strauss [4,*,†] and Anna Gotz-Więckowska [1,†]

1 Department of Ophthalmology, Poznan University of Medical Sciences, ul. Augustyna Szamarzewskiego 84, 61-848 Poznan, Poland; anetachoreziak@gmail.com (A.C.-M.); anna.czarnocinska@ump.edu.pl (A.C.-C.); agotzwieckowska@ump.edu.pl (A.G.-W.)
2 Department of Neonatology, Poznan University of Medical Sciences, Polna 33, 60-535 Poznan, Poland; dawidszpecht@ump.edu.pl
3 Department of Perinatology and Women's Diseases, Poznan University of Medical Sciences, ul. Polna 33, 60-535 Poznan, Poland; a.mrozikiewicz@ump.edu.pl (A.S.-M.); kdrews@ump.edu.pl (K.D.); gkurzawinska@ump.edu.pl (G.K.)
4 Institute of Human Genetics, Polish Academy of Sciences, Strzeszynska 32, 60-479 Poznan, Poland
* Correspondence: strauss@man.poznan.pl
† These authors contributed equally to this work.

Supplemental Results

Gene-Environment Interaction between the MMP-1 and TIMP-1 Genotype and ELGA

We observed that the associations between the *MMP-1* rs1799750 variant (in the entire group) and the *TIMP-1* rs4898 variant (in female newborns) with ROP became more pronounced after adjusting for confounding factors. This suggests that the results were influenced by the confounding factors under study. Consequently, we conducted a gene-environment interaction analysis involving *MMP-1* and *TIMP-1* variants, gestational age (GA), birth weight (BW), and gender.

These interactions were assessed using the method described by Botto and Khoury [39], which is based on the use of a multivariate table with a two-by-four layout. The assessment was performed using univariate analyses (Fisher's test). The Rothman synergy index (*S*), which indicates a deviation from the additive model of interactions, was also determined. The interpretation of the coefficient values is as follows: $S = 1$ indicates no interaction; $S < 1$ indicates a relative decrease; and $S > 1$ indicates an increase in the strength of the interactions between the two factors. Extremely low gestational age (ELGA; defined as GA < 28 week) and extremely low birth weight (ELBW, defined as BW < 1000 g) were considered.

We found that ELGA was a factor that interacted with the tested variants. This factor significantly elevated the risk of ROP, increasing it by approximately 9-10 times. The presence of the *MMP-1* rs1799750 1G allele in conjunction with ELGA was observed in 0% of children without ROP, but in 44% of cases with ROP (Table S1).

Table S1. The effect of gene-environment interaction between the *MMP-1* and *TIMP-1* genotypes, and extremely low gestational age (ELGA) on the incidence of retinopathy of prematurity (ROP).

**Risk Factors**		**Incidence of ROP**		**Statistical Analysis OR (95%CI); *p***
MMP-1 rs1799750	ELGA	I No ROP	II ROP	II vs. I
2G/2G	⩾28 wk.	15 (30)	4 (8)	reference
2G/2G	<28 wk.	4 (8)	11 (22)	10.3 (2.1–50.6); 0.005
1G/2G + 1G/1G	⩾28 wk.	31 (62)	13 (26)	1.6 (0.44–5.65); 0.553
1G/2G + 1G/1G	<28 wk.	0 (0)	22 (44)	155.0 (7.77–3092); <0.0001
OR expected from additive model (G+E) [a], Synergy index [b]				10.9; 15.6 ↑
TIMP-1 rs4898 (female)	ELGA	I No ROP	II ROP	II vs. I
TT	⩾28 wk.	4 (12)	3 (14)	reference
TT	<28 wk.	0 (0)	4 (18)	9.0 (0.34–238); 0.200
TC+CC	⩾28 wk.	19 (76)	4 (18)	0.21 (0.03–1.45); 0.132
TC+CC	<28 wk.	3 (12)	11 (50)	3.7 (0.47–28.4); 0.303
OR expected from additive model (G+E) [a], Synergy index [b]				8.1; 0.4 ↓

a-Expected OR = OR1 Observed (reference genotype and <28 wk.) + OR2 Observed (studied genotypes and ≥28 wk.) − 1; b-The Rothman synergy index (S).

Compared to children with the rs1799750 2G/2G genotype born at or after 28 weeks of gestation, children carrying the 1G allele born before 28 weeks of pregnancy had a 155-fold increased risk of ROP ($p < 0.0001$). The Rothman synergy index ($S = 15.6$) indicated a multiplicative interaction, signifying a relative increase in the risk. A similar effect was observed for the *TIMP*-1 rs4898 C allele in female newborns. This allele was found to be a protective factor against ROP. The co-occurrence of this allele with ELGA reduced the risk of ROP from 9-fold to 3.7-fold. However, this effect was not statistically significant due to the small size of the study group. The Rothman synergy index ($S = 0.4$) indicated a multiplicative interaction, signifying a relative decrease in the risk.

References

39. Botto, L.D.; Khoury, M.J. Commentary: Facing the challenge of gene-environment interaction: The two-by-four table and beyond. *Am. J. Epidemiol.* **2001**, *153*, 1016–1020. https://doi.org/10.1093/aje/153.10.1016.
